# Capability to make well-founded decisions: an interview study of people with experience of sickness absence who have common mental disorders

**DOI:** 10.1186/s12889-022-13556-4

**Published:** 2022-06-14

**Authors:** Christina Andersson, Annika Jakobsson, Gunilla Priebe, Mikael Elf, Robin Fornazar, Gunnel Hensing

**Affiliations:** 1 School of Public Health and Community Medicine, Institute of Medicine, Sahlgrenska Academy, University of Gothenburg, Gothenburg, Sweden; 2 Section of Health and Rehabilitation, Sahlgrenska Academy, University of Gothenburg, Gothenburg, Sweden; 3 Institute of Neuroscience and Physiology/Occupational Therapy, PO Box 115, 405 30 Gothenburg, SE Sweden; 4 Assessment team, Primary health Care, Gothenburg, Region Västra Götaland Sweden; 5 City executive office; City of Gothenburg, Gothenburg, Sweden

**Keywords:** Capability, Decision making, Sickness absence, Rehabilitation process, Common mental disorders

## Abstract

**Background:**

Sickness absence and rehabilitation processes can be challenging for an individual. At a time of generally reduced capacity, the individual must comprehend and navigate through several options. The aim of this study was to investigate the prerequisites for support, knowledge and information related to decision making experienced by people on sickness absence due to common mental disorders.

**Methods:**

A qualitative explorative approach was used. Face-to-face interviews took place with 11 sick-listed individuals with common mental disorders. Patients were recruited from different sources in the western part of Sweden, such as primary health care centres, patient organizations and via social media. Data analysis was performed using manifest content analysis, meaning that the analysis was kept close to the original text, and on a low level of interpretation and abstraction.

**Results:**

The analysis revealed three themes that described experiences of decision making during the sick leave and rehabilitation process: *Ambiguous roles challenge possibilities for moving on; Uncertain knowledge base weakens self-management*; and *Perceived barriers and enablers for ending sick leave.*

**Conclusions:**

Our findings suggest that alternatives need to be found that address sickness absence and rehabilitation processes from a complex perspective. Collaboration between stakeholders as well as shared decision making should be considered when the time for return to work is discussed with sick-listed individuals. Other factors in the context of the individual must also be considered. Current knowledge on strategies to improve health/well-being while being in the sick leave process need to be elaborated, communicated and adapted to each individuals’ unique situation, including clarifying rights, obligations and opportunities during the sick-leave process.

## Background

Sickness absence and rehabilitation processes can be challenging for the individual [1] because they involve decisions related to treatment, rehabilitation and return to work. At a time of health problems and generally reduced capacity, the individual must comprehend and navigate through several options presented by health care and social insurance institutions [2]. Decisions must be made, some of which could be life changing or at least critical in relation to a continued work life. Thus, the ability to make well-founded decisions is key during this process. However, studies on the specific needs of patients for decision making during sickness absence and the rehabilitation processes are scarce. More research exist on experiences of the sick-leave process itself including factors that facilitate or complicates return to work. Some recurring factors include different kinds of co-worker support [3, 4], a supportive employer-employee relationship [4], accommodation of work and work strain [4], help to maintain routines in life and work identity [5], professional support and coordination between different actors/systems [3, 4], help to improve mental health and foster self-efficacy and balance in life [4, 6], and ability to participate in one’s own sick-leave/rehabilitation process [6].

One of the few studies with a focus on decision making [7], a qualitative study of women with experiences of sickness absence, identified “feeling capable and having belief in one’s capacity” as important for making well-founded decisions. The study participants also expressed a need “to be sure” and they described that, initially, they were active in information seeking but became gradually less active because they had difficulties finding the information they needed. Finally, the study identified “a supportive context, commitment, respect and shared responsibility” as important for enhancing decision making. This study put forward that making well-founded decisions was not only a cognitive, intellectual exercise but also a multifaceted process including emotional and social aspects.

This aligns with a variety of theoretical models that emphasize the importance of understanding the (non) actions of individuals’ in their context. One such perspective, when discussing sustainable employability, suggested by Van der Klink et al. [8] is based on Amartya Sen’s Capability Approach [9], which illustrates how the individual’s capability to conceive, revise and exercise freedom (act in his or her own interest) is connected to how well their respective resources harmonize with surrounding institutions. Van der Klink et al. [8] further interpret the capability approach as follows: “Capabilities therefore represent a person’s opportunity and ability to achieve valuable outcomes, taking into account relevant personal characteristics and external factors: being able and enabled” (p. 74). An individual’s capability to make, for example, health promotive decisions is only partly determined by his or her collective resources (functionalities), because their usefulness is connected to how well they connect with the mandate, organization and performance of the societal institutions that govern the area of life in question. Thus, in a sickness absence and rehabilitation process, if understood from this perspective, it is important to focus not only on the individual’s resources, values, health status and so on but also on the existence of reciprocal links between these and for example, the support structures of the social security system.

This insight is of particular importance for people affected by common mental disorders (CMDs), because their health situation limits their ability to make the type of decisions that are needed for the use of societal services. Furthermore, trusting relationships with professionals is crucial to their experience of health care and social services, especially so when information about services is fragmented or inadequate or different stakeholders convey conflicting messages [10]. In addition, members of this group have experienced being excluded from decision making due to lack of knowledge and information and because it was felt that they were not capable of participating in a decision due to insufficient insight as a result of their illness [10].

Quality collaboration with, for example, health care staff is therefore an important building block, which the capability approach deems necessary, between an individual’s resources and the institution. Self-confidence and peace of mind can also be understood as functionalities. When they are lacking, the possibility to express needs, ask for more information or for new suggestions in vocational rehabilitation is reduced. The individuals do not feel that they are capable of participating in a decision when they do not have the whole picture [11, 12].

In Sweden, where this study was carried out, sickness insurance is universal and encompasses all citizens, and non-citizens with working permission. The first 7 days of sick-leave are self-certified while a medical certificate is needed from the 8th day. Sickness benefits are provided if the person has a reduction of work capacity with at least 25% due to disease, injury or medical symptoms. There is at present no limit for how long a person can be sick-listed. For persons with CMD the reduction of work capacity can be difficult to explain and physicians find it particurlary difficult to asses work capacity in CMD [13]. Another factor that has been raised as problematic in the Swedish system is collaboration between the Social Insurance Agency, the health care and the employer. Thus, the Swedish system puts high demands in particular on long-term sick-listed individuals to navigate rules and regulations and to communicate with several different professional representatives.

So far, few studies have explored the perceived needs of persons affected by CMDs during the sick-leave process [7, 14]. To strengthen their capability to use health care and social services effectively, we need to better understand how representatives of this group experience the conditions for making well-founded decisions during sickness absence and the rehabilitation process. This kind of knowledge can also be important for future interventions to prevent a prolonged rehabilitation process and shed light on possible interactions between important factors.

The aim of this study was to investigate the prerequisites for support, knowledge and information related to decision making experienced by people on sickness absence due to CMDs.

## Methods

### Design

A qualitative explorative approach was decided as the most appropriate method for the study. This enables individuals with CMDs to describe how they perceive their situation and how they make decisions during the rehabilitation process in their own words. Face-to-face interviews were considered the most suitable method for data collection, but potential participants were also offered focus group interviews. However, all participants chose individual interviews.

### Study participants and recruitment

Purposive sampling was used, and the inclusion criteria for participation in the study were that the participants should be between 18 and 65 years of age and have ongoing or recent experience of sickness absence from work or studies due to CMDs. People with severe depression or anxiety, or individuals who were suicidal were not invited, mostly for ethical reasons. We strived to capture as many different ideas and experiences as possible, therefore a variation in characteristics regarding age, sex, education, occupation, length of sickness absence and country of birth was desirable.

To recruit participants, we put flyers in the waiting rooms at primary health care centres and health care staff were asked to spread the word about the study when they met patients fulfilling the inclusion criteria. The health care staff were not reimbursed for their efforts. Flyers were also put up on pin boards at common venues (e.g. libraries, food stores) within Region Västra Götaland. We sent e-mails through a patient organization for people with mental health problems and an advertisement was posted on their webpage. Advertisements through official social media channels (Facebook and LinkedIn) and a webpage for the University of Gothenburg were also used.

### Interviews

The interviews were semi-structured, and a thematic interview guide was created by the research group, consisting of researchers with varied professional backgrounds and experience of insurance medicine. To capture relevant experiences for the aim of the study, the interview guide was constructed in line with the dimensions according to the Capabilities Approach [12]. The thematic guide was piloted in one interview, and no suggestions for changes were made. The interview guide consisted of four domains:

- the period sick-listed (e.g. how the days at the beginning, the middle and the end went down and how health and the ability to be active varied within the process)
- information regarding CMD and sickness absence
- the sick-listing process (e.g. support or lack of support from stakeholders, friends and relatives, and what worked well and not so well within the process)
- the path to recovery (regarding health and return to work), causes of CMD and sick-listing, and spontaneous ideas for web-based support.

The participants were interviewed at the University of Gothenburg (except for two telephone interviews). All interviews were conducted and audio recorded by CA (a registered occupational therapist and Doctor of Medicine) and RF (Master of Public Health) between June and September, 2017. One interview was transcribed from the interviewer’s memory immediately after the interview due to technical problems with the audio recording. The interviews lasted between 42 and 94 minutes.

Recruitment was originally planned to continue until theoretical saturation was achieved. However, the recruitment of participants went slowly, so when 11 interviews were conducted and the information was found to be relevant and rich in content, it was decided that the data collection could cease.

Of the 11 interviewees, all participants lived in urban areas. The background of the participants varied as anticipated (Table 1). They were employed within both public and private sectors and involved in both operative and administrative tasks (e.g. mailman, nurse, and engineer). At the time of recruitment, 10 of the participants were on sick leave, but some had been sick-listed partly, full time or a variation of this over time. One participant was no longer on sick leave but had started an educational course. In addition, the participants had different experiences of the sick leave process depending on the length of the sick leave spells.

**Table 1:** Sociodemographic description of the participants (*n* = 11)

ID	Gender	Age (years)	Education	Length of sick leave	Currently on sick leave
1	Female	34	University	3 months	No
2	Female	41	Secondary school	2 months	Yes
3	Female	33	University	15 months	Yes
4	Female	55	University	5 years	Yes
5	Male	53	Secondary school	10 years	Yes
6	Female	44	University	3.5 years	Yes
7	Female	20	Secondary school	3 months	Yes
8	Female	33	University	2 months	Yes
9	Female	52	Secondary school	2 years	Yes
10	Female	54	University	7 years	Yes
11	Female	34	University	3.5 years	Yes

### Analysis

A manifest content analysis inspired by Graneheim and Lundman [15] was performed, which means that the analysis was kept close to the original text (the transcribed interviews) and on a low level of interpretation and abstraction.

The interviews were transcribed verbatim by a professional agency and two of the authors (CA and RF) from the multidisciplinary team began the analysis. They each read the transcripts to gain a common understanding of the whole. Then, the interviews were read line by line to identify meaning units related to the aim of the study. The meaning units were compared and a high consistency was found among the chosen meaning units. Differences were discussed, and after a second review, the meaning units were decided and grouped according to the four domains in the interview guide.

In the next step, conducted by two of the authors (CA and AJ, a registered nurse and Doctor of Medicine), the meaning units were condensed and coded. The codes were regrouped according to their content, and each group with similar content constituted a theme. The themes were scrutinized to make sure that no data related to the aim were missed or excluded. Based on the themes, categories and subcategories were identified. The themes, categories and subcategories were then labelled.

One author, ME (a psychologist and PhD) analysed the meaning units connected to the domains in the interview guide. These were first merged into larger units and then merged into three overarching themes. The concordance between the results of the two analysis procedures was good, and the final results were agreed upon after a critical review by the entire research group.

### Ethics

Due to the sensitive nature of the topic, efforts were made to handle the interview situation with care. All participants were informed about the purpose of the study, that participation was voluntary and that it was possible to leave the interview at any time without justifying why. The participants signed an informed consent form before starting the interview. All participants completed the interview. The Regional Ethical Review Board in the University of Gothenburg, Sweden (Dnr 810–16), approved the study.

## Results

We identified three themes from the interview material that captured the participants’ perceptions of the interaction between the person on sick leave and representatives of the institutions involved. These three themes describe three aspects of the sick-leave and rehabilitation process. The first theme, *Ambiguous roles challenge the possibilities for moving on*, focuses on the factors that the respondents described as relevant for moving forward in the process. It includes a duality related to perceptions of one’s own responsibility and not having enough energy for participating in important decisions. The second theme, *Uncertain knowledge base weakens self-management*, describes the quality of information exchange as something necessary for good governance of the process. It concerns both the experience of information not being presented or clarified, and uncertainty about how one’s own statements were received. The third theme, *Perceived barriers and enablers for ending sick leave*, includes expressed needs for external support as well as the use of personal characteristics to take a constructive part in the sick-leave process (Table 2). Citations are given to illustrate the subcategories and ensure an empirical foundation [16], and labelled according to sex, age and length of sick leave.

**Table 2:** Overview of the results

	Themes		
	Ambiguous roles challenge possibilities for moving on	Uncertain knowledge base weakens self-management	Perceived barriers and enablers for ending sick leave
Categories	The influence of demands and responsibilities	Understanding available information to make healthy decisions	The importance of time
	The significance and authority of the physician	Deficiency of knowledge	Managing everyday activities
	The impact of roles in the workplace		The importance of support and recognition
	The need for coordination and continuity		Personal characteristics

### Ambiguous roles challenge possibilities for moving on

This theme embraces the respondent’s perceptions about their own role in the sick-leave process. In addition, it captures how they viewed their relationship with representatives of, for example, the Social Insurance Agency, health care and the workplace. It includes a duality related to perceptions of one’s own responsibility and not having enough knowledge or energy to participate in important decisions. In particular, the need for coordination was mentioned when respondents discussed the interaction between different stakeholders, especially when it did not develop satisfactorily, or in a way that made sense to the respondents.

#### The influence of demands and responsibilities

The participants described that they took, and wanted to take, personal responsibility for making things happen during the sick-leave process, yet underlined that this often demanded more effort and energy than they felt they could spare. Such demands were sometimes experienced as being due to lack of understanding or knowledge from the professionals,

… When you are feeling bad you still have to … keep nagging and calling and you get angry … or not angry, but annoyed, and you have to use so much energy even though you really do not have the strength. (F, 41, 2 months)

The respondents put forward that they felt that they were expected to take wide-ranging responsibility for their own sick-leave process. This perceived responsibility to be active weighed heavy on them and was described as a source of worry over, for example, not being able to give the correct health information or suggest the most relevant intervention to the physician.

And if you have to phone at a certain time, then you have to do it. On Monday they put me on hold. … So, I sat between eight o'clock and five, a full day … I decided, I have to get hold of these people. And the doctor said it, "yes, you are stubborn when you want." But that's not good. (F, 54, 7 years)

A certain amount of energy and strength seems to be necessary, and even expected in the sick-leave process. The participants also indicate that lack of this functionality is not neutral in the sick-leave process, but something that consumes energy, and therefore, could hinder the process from moving forward.

#### The significance and authority of the physician

The interaction with physicians was regarded as key to the process because many actions depended on the physician; for example, communication with the Social Insurance Agency when representing the patient and writing a correct and informative sickness certificate. However, worries were expressed about the capacity to convey problems in a way that truthfully reflected one’s health situation. The encounter with the physician was sometimes perceived as an occasion where they had to display their collective strengths, be sharp, and do their best.

Because the doctor's appointment is an achievement, you kind of have an adrenaline rush during that meeting; so in that situation, it may be difficult for the doctor to see how bad you actually are. (F, 33, 15 months)

Lack of continuity of physicians (e.g. because of temporary positions and changes) was experienced as complicating and delaying the process, and led to concerns about not being fully understood. Seeing a new physician at each appointment made it necessary to tell the same story repeatedly, reducing the options for discussing plans for the future.

But especially in the beginning, when there were new doctors every time. It felt like you had to repeat yourself; you had to tell the same story over and over again; you had to get to know a new person. (F, 34, 3.5 years)

These concerns about not being fully understood led to feelings of uncertainty in relation to the next visit and to stressful feelings of having to formulate strategies for how to get a new certificate as an alternative to being forced to go back to work. This was further amplified when communications about the rules for being on sick leave and getting back to work were experienced as unconstructive. In some situations, the physician’s communication style was even perceived as threatening.

So after four months, you know, she was frantic, more or less. "Yes, but … You have to start working now". But I cannot work. (F, 44, 3.5 years)

The capacity to repeatedly, efficiently and convincingly portray problems and health status to the physician was described as a key functionality. In addition, the quality of the relationship was described as being potentially hampered by organizational limitations, such as frequent changes of staff and the extra explanatory burden it put on the individual.

#### The impact of roles in the workplace

The importance of the workplace in managing the sick-leave process was described in several ways related to roles that appear while being at work. Support from colleagues and managers contributed to their active participation in planning for return to work. Regular meetings with a manager induced a feeling of being a valuable employee and acceptance of special needs connected to the health situation; for example, a need for less social interaction was highlighted as important for decisions connected to work adjustment.

In contrast, absence of strategies by management or their lack of knowledge about the ways to support and adjust for the employees return to work was described as a challenge. Examples include lack of planning, unclear organization or distribution of responsibilities related to the return to work. Such uncertainties led either to disappointment over promises not fulfilled or that the person on sick leave had to remind the employer about how to create conditions for return to work. One woman looked forward to starting return to work training; however, the conditions were not the best for her. She explained:

… Not at my usual workplace; it was at another place. And I crashed again, after … my six hours a week became way too much. (F, 44, 3.5 years)

Thus, when the workplace was experienced as a safe and well-known arena, with support from employer and staff, this became an enabling factor during the sick-leave process. However, when too much responsibility was placed on the person involved, or when there was a lack of interest and knowledge about how to support a sick-listed employee, the effect was the opposite.

#### The need for coordination and continuity

The need for coordination of different interventions was expressed as central for planning to return to work. Insufficient information and contradictory messages from health care professionals caused confusion for the sick person. Interventions both within the health care system and between different stakeholders were not always synchronized, and/or planned for but not executed. Examples included care measures not given at the right time, or when there was a lack of consensus about regulations for sick leave, leading to insecurity, worries and anxiety. Another perceived problem was that several promises were made that were never fulfilled, as quoted below:

But I have not received any information on how to handle the stress. Because at first they thought that I should talk to a counsellor, but then it never happened. And I do not know why. The health centre … . they said that occupational health care will take over my care and then occupational health care said “We cannot take you over” … (F, 41, 2 months)

This category also includes statements about being the most important coordinator or being completely left out of the planning process. Uncertainty about getting the best treatment and the possibility of taking an active part in the planning were also discussed.

You do not really dare to speak out. There are different people; it feels like there is no coordination, not even within their own house. They say they work in teams, but it does not feel like the team always has all the information. (F, 34, 3.5 years)

On the other hand, coordination and consensus regarding the application of rules and about suitable interventions led to a feeling of security and an overview of what to relate to during the sick-leave process.

But it feels like you should get more information but maybe just suggestions about getting help from a rehab coordinator. Because if you are paralyzed by anxiety and probably depression as well, you will not be able to talk to any of them [The Social Insurance Agency]. (F, 33, 15 months)

The participants expressed the need for synchronization between the individual’s specific situation and the possibilities for support within the health care and social security systems. This could further be interpreted as need for bridging between the individual functionalities and the contextual capabilities.

### Uncertain knowledge base weakens self-management

Within the second theme, thoughts and experiences on information were discussed. Information comprised both the ability to make sense of the information that was given and that the best information concerning the individual situation was available. Lack of knowledge for the individual as well as caregivers and other stakeholders emerged. The individual experienced lack of knowledge about making decisions on possible interventions and self-care. On the other hand, the professionals did not appear to understand all the dimensions in the return to work process and measures needed to support this process.

#### Understanding available information to make healthy decisions

The participants stated a desire to gain and search for more credible knowledge concerning health issues and matters related to the sick-leave process. Web sites were used as a source of knowledge, as well as well-informed friends or acquaintances. On the other hand, worries were expressed about what information could be trusted. Another hesitation concerned the participant’s own ability to perceive and comprehend the information. In some situations, the strength to search for information was lacking.

… I feel that I have missed something; this is it “How do you deal with anxiety?” … . I do not believe in CBT [cognitive behavioural therapy]. But, somehow CBT is done in Sweden, because the National Board of Health and Welfare says so. Yes, but show me that it's good. I can read research reports. I do not have difficulty reading information … but I do not have the strength to search for it. (F, 44, 3.5 years)

Uncertainty due to unclear information about rights, obligations and opportunities was expressed. Not knowing what the next step is as the basis for decisions and who is responsible for these decisions and actions led to insecurity.

… It can take several years to come back. And I might have wanted to know that information from the beginning. First, being put on sick leave for three weeks does not mean you're healthy in three weeks. “We will probably put you on sick leave for much, much longer, but I want to get in touch with you at regular intervals.” (F, 34, 3.5 years)

Clearer and more transparent information was asked for, such as a schedule for actions and how to prioritize these. This was related both to information about the sick-leave process and about options for treatment. The informants also felt insecure about how their information about the situation was perceived and handled.

The capacity to become an active participator in your own process was discussed with a focus on information as a transformative tool, connecting the person with the surrounding context. When such information was not communicated, the possibilities for cooperation was hampered.

#### Deficiency of knowledge

Representatives from the health care system, including physicians, do not explicitly inform about the options available for the person on sick leave. Instead, the person is asked about his or her preferences without knowing the alternatives or the effect on return to work.

… they have asked me what help I need. It is very difficult when you do not know what there is to choose from. I do not know; this information does not appear anywhere. (F, 20, 3 months)

The individuals on sick leave needed information that they could rely on concerning the different options, but they also needed support or guidance in how to implement the chosen actions step-wise adjusted for themselves.

One thing that I think has been my main problem is that you have been told that exercise is great. … Mm, great. But what type of exercise? I could not work that out myself. I just want "now you have to do this". (F, 34, 3.5 years)

Another knowledge deficiency concerns paying attention to early signs of illness and risks for sick leave. Examples were given about trying to solve the situation while struggling to stay at work, leading to a total breakdown before seeking health care.

The great difficulty and lesson is not to ignore signals. And that's probably the hardest part. You notice that with people who “are” their working lives and so may not be on sick leave yet. But I can see quite strong signs or symptoms telling me that it is starting to be, you know, a risk zone. (F, 52, 2 years)

Not being able to understand and describe one’s shortcomings led to an inability to start making necessary adjustments to the new circumstances, both in the workplace and outside work. The perception of one’s own ability was sometimes overrated, both before and during sick leave.

And limitations and … or not limitations, but values, have been incredibly strong. I have been doing just as much at home, with the children, driving and going here and there. And all the projects we have to do in the house and so on. In hindsight, I can see that it was doomed to crash. (F, 44, 3.5 years)

A lack of knowledge also occurred in other important instances during the sick-leave process. Shortcomings were described, both in the health care system and the Social Security Agency, concerning assessment of work ability, especially when factors at work and in everyday life coincide in influencing the ability to work. Shortcomings also appeared within the employer in adapting the work situation to the individual’s need for change and support.

… that they get a really proper reminder about rehabilitation responsibilities, what is important to think about, what … how you have to try to create conditions for this person to get back to work. And I was never offered job rehabilitation in that situation. This was one thing … it was actually an important thing that I never understood. Because I should not have worked. I should have been rehabilitated because I had no idea what I was or was not capable of. (F, 33, 15 months)

In this category, the need for new insights and new knowledge, from both lived experiences and professional expertise, became apparent. In other words, there is lack of knowledge in order to support the capabilities, both whitin the individual, as well as in the surrounding systems.

### Perceived barriers and enablers for ending sick leave

This theme comprises statements about the ability to adjust to the new situation. The statements focused on both external and internal factors that were described as having a positive or negative impact on the process and the ability to return to work.

#### The importance of time

Time was an important factor for recovery. Time was described as important for regaining a sense of one’s capacity, and too much pressure and demands was experienced as counterproductive. Furthermore, the sick leave had to include meaningful activities to have a positive impact on the recovery process. One woman explained how she needed time during her sick leave:

Time has been, again, a very important factor. To get … proper time to get back to who you are. So at first, it was very much about sleeping. (F, 34, 3.5 years)

She also highlighted:

I feel that even if I'm not completely healthy, I do not know if I will be healthier if I go home. And I need something to do with my time that I feel is meaningful. (F, 34, 3.5 years)

Time was also considered as important for learning new aspects about one self. This was exemplified as learning how to handle the impact of illness, how to set limits for commitments, and how to speak up for one’s rights. To learn how thought patterns influence behaviour and everyday life was seen as part of the change towards recovery.

… a struggle every day to learn, to develop oneself, to get to know me. What are my challenges? What am I lacking? Or lack? Yes, but then what characteristics and behaviours and thought patterns do I have that affect how I behave? (F, 44, 3.5 years)

Another example was the ability to make new plans, for example, change of workplace.

But I have been too cowardly to resign from a permanent job. But now I feel it is not possible to work and I cannot sacrifice my health to work as I do … So this has taught me that you have to dare … (F, 41, 2 months)

The respondents spoke about time as an enabling factor for changing inner resources. Having enough time during sick leave was also important to actually fulfil commitments, such as carry out treatment programs.

#### Managing everyday activities

To manage everyday activities was described as an important factor on the road to recovery. One positive aspect of managing everyday activities created structure in everyday life while not able to work. Maintaining everyday activities, such as having a family role and social contacts, or taking part in some work activities, created the feeling of being an able person and not just being sick.

It is very good to be out and work when you have depression, because you are forced to meet people. Even if you work only for a few hours, not full time, you meet people. So it's not really the best thing in my opinion to be on full time sick leave, because it's easy for you to just lie down and sleep and not get out. (F, 34, 3 months)

Allowing oneself to take part in activities that were enjoyable, such as skating, taking a walk in the woods or engaging in your personal interests, was experienced as health promoting. On the other hand, some had difficulties allowing themselves to have fun, because it led to a bad conscience and a feeling of slipping away from responsibilities such as return to work.

I cannot do anything fun, because then I “play hooky”. (F, 34, 3.5 years)

The participants also talked about how some everyday activities, including family relationships, were experienced as a strain when trying to focus on returning to work. This was exemplified as having the responsibility for children with special needs.

… Several of my children have special needs; this adds to the work involved in having children and taking care of a home, and than to be good enough about that … . (F, 52, 2 years)

Not being able to manage everyday activities could also be seen as an indicator of the individual’s capacity to cope with self-care matters, and even less ability to work.

The Swedish Social Insurance Agency is forcing me to return to work as soon as possible, but I can not even cook for myself yet. (F, 33, 15 months)

These statements on the importance of everyday activities reflect the reciprocal relationship between inner capacities and contextual factors. External possibilities can lead to a stronger sense of self-efficacy, whereas the opposite can lead to stagnation in the recovery process.

#### The importance of support and recognition

The participants emphasized the importance of support from partners, relatives and significant others during the recovery process. The support involved being helped with practical matters, such as self-care, housework and planning tasks, as well as emotional support and understanding of the circumstances. Encounters with people in similar situations increased the feeling of not being alone, which was beneficial in their situation.

The fact that you feel that you are not alone, you hear about other people's problems, and maybe hear how they managed, it allows me to handle my own problem, in a different way. … If you then have problems with resources, okay, then think that you should collect a few ideas instead of just from one person … . I met a very nice person at physiotherapy, and I now have contact with them and I can talk to them. We do not have the same diagnosis but we still understand each other's situation. (F, 34, 3.5 years)

One factor that emerged from the participants’ experiences of professional support was the importance of being listened to and that someone believed in their story. This mutual respect was vital for achieving recovery. The participants related issues about how their situation and inabilities were perceived and described in the sickness certificate, and uncertainty about whether you had fulfilled the conditions according to the sick-leave rules. Both private and professional support could also consist of practical help and support with daily activities, as well as planning the recovery process in small steps and making individual adaptations to proposed measures.

#### Personal characteristics

The participants’ personal characteristics were described as both benefits and hindrances, exemplified by being stubborn, sustained, and having high demands. Inner traits such as stubbornness or sense of duty were described as supportive for achieving one’s commitments at or out of work, or having the energy to engage in their own process.

So I am stubborn as sin [laughs] and I place very high demands on myself. I want to be good so that I will return to being a part of society. (F, 34, 3.5 years)

On the other hand, personal characteristics such as high demands, passivity or lack of a driving force could work in the opposite direction.

Yes, but it may be I have … it may be a negative self-image, or self-esteem, and as well as passivity or lack of drive or difficulty in starting … difficulties with initiation. (F, 52, 2 years)

Personal characteristics sometimes played an important role in the ability to adjust to a new situation. However, the impact of these qualities did not work in isolation, but appeared and were adjusted depending on the context.

## Discussion

The participants in this study perceived that information and knowledge are essential factors for decision making regarding health issues related to the rehabilitation process. The study also showed that a supportive workplace, as well as a person-centred approach from professionals, could prevent a prolonged rehabilitation process, and facilitate the person’s own participation in planning for returning to work. However, the findings revealed three themes that highlight the complexity of how other important factors influence the process. Ideally, when roles between the person and professionals are clearly defined, adequate and relevant information is communicated, and the person has a supportive environment, the process can move forward. When obstacles arise in any one of the three themes, return to work can be delayed, and the lack of coordination can put high demands on the person.

### Findings in relation to previous studies

Previous studies have shown that the concept of health literacy can be important in understanding underlying factors for making well-grounded decisions [7]. Just as with definitions on health literacy [17], our findings show that this is not only a matter of obtaining, perceiving, analysing and valuing information. Making sense of information also relies on the context, for example, where and how this information is delivered and adapted to the actual situation of the sick-listed person. In our study, participants spoke of normally being a capable person but for the moment losing the ability to obtain and make sense of information. The context also matters in the sense that, in contrast to being in a familiar situation as a competent worker, being on sick leave is a completely new situation, where previous knowledge cannot be applied. Our findings also correspond to another aspect of health literacy, underlining the importance of the organization [18]. In this sense, the organization contributes to whether or not the person experiences enough support and information, for example, by how well the planned measures are adjusted for the person on sick leave. The question remains whether deficient knowledge on how to move forward in the process lies within the individual or the organization. According to our findings, this probably applies to both.

Our results could also be discussed in relation to the concept of empowerment, which has been described on an individual level, as well as on an organizational and community level [19]. Empowerment has been used to explain factors concerning the situation while being in the rehabilitation process [20], highlighting the importance of the individual level, where the interaction between the person and the professional has been suggested to be an important factor. This can lead to either positive or negative self-evaluation, with an impact on what is called psychological empowerment or disempowerment [21]. This could be exemplified in our study, whereby uncertainty about roles and contradictory information were expressed as leading to worries and insecurity about the next step. Empowerment is further defined as taking part in your own rehabilitation process, and also having a sense of control, as well as actual control over daily life. Such control is not only dependent on inner psychological factors but also on contextual or structural factors, such as having access to support and knowledge, and participation in meaningful activities [22]. In our study, the participants expressed the importance of managing ordinary, everyday activities as a road to recovery, as well as support from important people around them.

Skoglund et al. [23], in their study on patients on sick leave, found that understanding that things change, and change takes time, was important. This can be compared with our study where the need for time was found to have a role to play in recovery while learning about oneself and finding strategies to change the work situation. Filling the time with structure, meaningful occupations and support from others have also been described in other studies [23]. Also, the timing of return to work actions is an important factor highlighted by the participants in this study. That a mutual decision on this has been made, for example, between the health professionals and the patient, is crucial and coincides with what Corbiere et al. [24] found in their study on decision making and return to work.

Achieving increased work capacity, and thereby shortening the sick-leave process, is a matter of interaction between finding strategies at work as well as in other everyday activities and has been highlighted in another study on interventions for return to work [25]. The authors discuss that awareness of one’s own capacity and self-reflection are promoted by breaking the traditional split between a focus on work life on the one hand and other aspects of everyday life on the other. Similar themes have been discussed in other studies proposing that rehabilitation efforts need to be individualized [14], taking both the individual level and social environment into account [26].

Such conclusions can be related to one of the categories in our study, deficiency of knowledge, where both participants and professionals had difficulties in interpreting and describing early signals, risk of sick leave, and the relevant interactive part of coping mechanisms in work as well as other everyday contexts. Taking the whole-life situation into account in the rehabilitation process could provide new insights into factors that promote more sustainable coping strategies [25]. Other findings under the same category concerned non-transparent information from, for example, the health care system about the measures available, how to get access to such interventions, and the timing and extent for such proposals. Too much was left to the individual to decide, with the risk of not being able to make a decision, or choosing and doing too much. A better understanding of the different factors that contribute to the potential for increased return to work could be warranted [27, 28]. Engaged professionals need to provide just the right information and planning related to the individual situation, and this could also be discussed in relation to ethical considerations [29]. The authors propose that different ethical considerations and cultural differences between stakeholders in the rehabilitation process could help explaining the deficiencies in, for example, mutual planning for the best measures that will support the individual. Such an approach could help explain the lack of communication between stakeholders, highlighted in a study [30] on different types of debate and information exchange between stakeholders, leading to unnecessary uncertainty in making decisions, both among professionals and the person on sick leave.

Last, but not least, the findings in this study correspond to the capability approach applied to the sick leave situation, describing the complexity between work ability and health [8]. Several categories could be summarized as describing the interplay between capabilities and functionalities in both the individual and in the surrounding context. To be highlighted, two categories stand out as something not previously observed or elaborated. One is the need for a coordinative effort as a bridge between inner and outer capabilities. The other is the need for adding new knowledge in order to understand the interplay between capabilities even better.

### Strengths and weaknesses of the method

In general, trustworthiness can be suggested to be relatively high in the study. The variation among the participants in the study was acceptable in terms of age (20–55 years) as well as the length of sick leave (2 months to 10 years), leading to a variability in different exeperiences during sick leave. In terms of education, participants were relatively highly educated; most had a university education and others had upper secondary education. This might have affected the participants’ work situation, and thus the ability to adapt the return to work process in a positive way, as well as the person’s requirements for information during such a process. The problems described by the participants affected the result, however, it was difficult to establish in what way. The participants were mainly women, which is in line with the fact that there are more women than men on sick leave with CMDs, although a gender balance would have been desirable. All researchers participated in the analysis via a so-called peer check through intersubjective dialogue. In addition, age, sex and professional background varied among the researchers, which contributed to a variation in perspectives of the phenomena under study.

We do not believe that the participants were damaged in any way due to the study. The overall purpose of studying experiences of information and support in people with CMDs and sickness absence was to contribute to future improvements of the situation. In addition, before the interviewees shared their stories with the researchers, they were informed about their rights. Two interviewers with different experiences conducted the interviews. They were in continuous contact to maintain the same approach and quality, including indicating to the participants that their experiences were important and taken seriously. Furthermore, the participants were invited to contact the interviewers and other responsible researchers with any questions regarding the project or their own participation, but nobody took that opportunity.

One advantage in this study was that the participants shared their experiences in an open and honest way, which yielded rich data. The broad competences of the research group increased the possibilities for data to be handled in a skilful and ethical manner.

## Conclusions

Our findings suggest that alternatives need to be found that address sickness absence and rehabilitation processes from a complex perspective. Collaboration between stakeholders, and consideration of factors in the context of the individual need to be expanded to improve the situation for sick-listed people. Current knowledge of strategies to improve health/well-being while being in the sick leave process need to be elaborated, communicated and adapted to each individuals’ unique situation, including clarifying rights, obligations and opportunities during the sick-leave process. One way of consolidating these needs could be through patient education with a focus on the complexity of being on sick leave, instead of coping strategies related to the specific diagnosis only. Such interventions could be accomplished both face to face and online.

Better understanding of knowledge on how to detect and describe early signs of activity limitations related to the capacity to work is needed. Further research on the effects of timing and interaction of different actions supporting the rehabilitation process could contribute to more substantiated decisions concerning sick leave.

## Data Availability

The data analysed during this study are not publicly available due to concern for the participating respondents but are available from the corresponding author on reasonable request.

## Abbreviations

CMD: Common mental disorder
SIA: Social Insurance Agency
CBT: Cognitive behavioural therapy
